# The First Inventory of Sardinian Mining Vascular Flora

**DOI:** 10.3390/plants14081225

**Published:** 2025-04-16

**Authors:** Maria Enrica Boi, Marco Sarigu, Mauro Fois, Mauro Casti, Gianluigi Bacchetta

**Affiliations:** Department of Life and Environmental Sciences, Centre for Conservation of Biodiversity (CCB), University of Cagliari, Viale Sant’Ignazio da Laconi 13, 09123 Cagliari, Italy; mariae.boi@unica.it (M.E.B.); mfois@unica.it (M.F.); maurocasti@hotmail.com (M.C.); bacchet@unica.it (G.B.)

**Keywords:** environmental pollution, environmental restoration, mediterranean vascular flora, metallophytes, phytoextraction, phytoremediation, phytostabilization

## Abstract

Mining activities and associated waste materials pose significant environmental challenges, including soil, water, and air contamination, along with health risks to nearby populations. Despite the harsh conditions of metal-enriched soils and nutrient-poor substrates, certain plants known as metallophytes thrive in these environments. This study examined the vascular flora of Sardinia’s abandoned mining sites, with a focus on identifying metallophytes and their potential role in phytoremediation. A comprehensive floristic checklist was compiled using literature, field surveys, and herbarium samples. Of the 652 *taxa* identified, 49% were metallophytes, with the majority categorized as facultative species. Notably, 27% of metallophytes were identified as suitable for phytostabilization, while 20% showed potential for phytoextraction. This study also highlighted the presence of endemic and endangered species, emphasizing the need for conservation efforts. The findings suggest that native metallophytes could play a key role in the ecological restoration of mining sites, though careful consideration of invasive species is necessary to avoid ecological disruption. This research provides valuable insights into the biodiversity of Sardinian mining sites and the potential for sustainable remediation strategies using native plants.

## 1. Introduction

Mining areas and the related mine waste materials represent a significant source of environmental contamination, and at the same time leave landscapes with evident scars (open pits, dumps) and present a health hazard for local inhabitants [1,2]. Mining remains, particularly open dumps, tailing dams, quarries, or accidental release of mine waste are the main sources of metal(loid)s in the surrounding environments, especially those with fine granulometry, like muds and fine sands, which can be easily subjected to aeolian dispersion and water erosion [3]. However, equally important are areas naturally enriched in metal(loid)s, where their concentrations are often well above the threshold limits established by national policy, as has already been observed, for example, at Sardinian mine sites [4,5].

Mine waste also limits the ecological spaces available for plant species and for the establishment of natural vegetation. These consequences are due to the presence of important concentration of metal(loid)s, the absence of topsoil, the poorly developed structure of the substrate, and the lack of nutrients (particularly K, N, and P) and organic matter [5,6,7,8]. These conditions, as well as the frequent instability of the substrate, prevent pedogenesis. In particular, some metals like Zn, Pb, and Cd have toxic effects on plant development. Even though Zn is an essential micronutrient for plants that plays an important role in various metabolic processes [9,10], its toxicity (ca. 100–500 mg/Kg) is manifested by chlorosis of new leaves and depressed plant growth [11]. Lead is a toxic element for plants and living organisms and affects many processes such as photosynthesis, mitosis, and water absorption [12,13]. The most common macroscopic evidence of Pb poisoning in plants are dark green leaves, wilting of older leaves, stunted foliage, and brown, short roots [13]. Cd is highly toxic to living organisms [14]. The main symptoms of Cd toxicity in plants are chlorosis, shunted growth, and plant necrosis [14,15,16]. Cadmium affects plants by inhibiting carbon fixation, decreasing chlorophyll content, inhibiting photosynthetic activity [17], and inducing overproduction of ROS [18].

In the Mediterranean Basin, several studies of plant diversity and their potential use for phytoremediation have been carried out [19,20,21,22,23,24,25]. These studies highlighted that, despite these unfavorable conditions, mine environments host several *taxa* able to colonize these substrates and reaching high level of vascular plant diversity, including many endemic species [7,19,24,26,27], like *Erica andevalensis* Cabezudo & J. Rivera, which grows only in the Iberian Pyrite Belt area (Spain and Portugal) [20,21], or *Limonium merxmuelleri* Erben subsp. *merxmuelleri*, which is exclusive to the metalliferous ring of South West Sardinia [19,28]. Plants which grow on these substrates, generally called metallophytes, have developed an intrinsic resilience to metal(loid)s stress and the abovementioned conditions [29]. Metallophytes can be obliged, in that they live and thrive only on metal(loid)-enriched substrates (polluted or natural), or facultative, in that they can be found growing in unpolluted or metal(loid)-enriched substrates [5,30]. Metallophytes are just one face of the wide and complex concept of edaphism, the geo-ecological relationship between the prevalence of endemic species and special edaphic conditions, together with gypsophytes, serpentinophytes, quartz-island–phytes, and dolomitophytes [31,32,33,34].

In the Mediterranean Basin, several abandoned mine sites, including their waste materials, have been left to exposed to the weather, often without reclamation, causing several issues in terms of human health and metal(loid)s pollution of the hydrosphere, pedosphere and biosphere [7,8]. Generally, pollution levels are reflected by the floristic composition of the region: on poorly consolidated materials with high concentrations of heavy metals, annual or perennial meadows can be observed, which will be gradually replaced by increasingly more evolved formations such as garrigues and maquis when weather agents wash away the substrate, the heavy metal concentrations decrease, and pedogenetic process begins [7,19]. Mining environments have intrinsic resilience [35] due to the interaction of different aspects: the interaction between the geosphere and biosphere at the interface of surface and groundwater takes place at the hyporheic zone [36] and can lead to the development of natural chemical processes related to the attenuation of metal content. Also, the biosphere contributes, for example, pioneer plants are able to grow in deeply polluted sediments due to their ability to adapt [37,38,39]. Indeed, plants have the ability to remove trace metals from water through different processes like biological uptake, surface adsorption, and the formation of biominerals that can lead to a decrease in the bioavailability of metals [38]. Direct and active intervention to start or accelerate the recovery of degraded environment like mining contexts has been suggested, and several studies emphasize both the benefits of spontaneous succession and the negative aspects of technical reclamation projects, including their high costs [40]. Generally, projects with a high level of human intervention can be inappropriate, because they can compromise the efficient restoration of post-mining environments [41]. On the other hand, unassisted, passive, and assisted revegetation of mining environments can promote adsorption of metals from the substrate and improve their removal and retention through plant uptake [7,42]. A vegetational and multitemporal landscape analysis of land cover transformation in the mine district of Monteponi (SW-Sardinia) from 1955 to 1998 [43], showed that a passive approach led to evolution of the natural vegetation, with also the presence of rare, endemic and endangered species. Indeed, in mine dumps the main transformations are towards maquis, garrigue, and woods.

There are several technologies that are suitable for remediation, using physical, chemical, and biological approaches [44]. Physical-mechanical technologies imply excavation or handling of the substrates and could move the pollution if it is not efficiently disposed of [45]. Chemical-oriented technologies need to use large quantities of reagents (i.e., soil washing) and can be applied in small contexts [46]. Hence, the application of these kinds of technologies is unsuitable in wide polluted areas because they induce modification of the landscape and soil properties and have high implementation costs [47]. When the amounts of polluted materials are widespread and plentiful, biological technologies are a viable solution for remediation. Among a wide range of biotechnologies, phytoremediation and bioremediation are the most supported by the scientific community [3,46]. In detail, phytoremediation is solar-driven and well adaptable to local conditions and can be aided by the implementation of substrate amendments and/or augmentation with microbial strains [46].

Metallophytes may modify rhizosphere conditions, as the availability of metals in the substrate around roots is strongly affected by root exudates [48,49]. In hyperaccumulator species, roots can improve metal bioavailability in the rhizosphere through the secretion of protons, organic acids, phytochelatins (PCs), amino acids, and enzymes. Excluder plants restrict transport of metals to the epigean organs and maintain relatively low metal concentrations in the areal parts over a wide range of soil metal concentrations. This behavior is made possible by the restriction of metals from entering the plant due to the absence of an uptake mechanism, or by the influence of root exudates that reduce the bioavailability of contaminants [6].

Among the different applications of phytoremediation, the most important are phytostabilization and phytoextraction, which use tolerant and accumulator/hyperaccumulator species, respectively [7,50,51]. Phytostabilization is a viable solution when the pollution is widespread and is suitable for protecting substrates from weathering, for creating a long-term plant canopy, and for reducing the visual impact of excavation and mine waste accumulation in dumps [3,7,52]. On the other hand, phytoextraction is mainly devoted to the economic recovery of metal(loid)s and for application in phytomining [53].

Within the framework of remediation of metal(loid)-polluted sites, a deep knowledge of local flora is desirable, as well as a focus on the endemic and alien components. Indeed, nowadays the use of native *taxa* is recommended for several reasons: these plants (i) are well adapted to local climate and substrate conditions [54]; (ii) favor micro-niche formation; and (iii) improve substrate fertility and permit the establishment of other species in the long term [5,55,56,57]. Despite evidence of the usefulness of numerous alien *taxa* for phytoremediation (e.g., *Arundo donax* L.) [58], they may pose a potential risk for local biodiversity, especially invasive *taxa*.

Sardinia has a long history of mining activities dating back to prehistoric times, which has played a significant role in shaping its landscapes and ecosystems [19,28]. Numerous studies have shown that, in Sardinia, there are numerous metallophytes that have specifically adapted to thrive in environments with high concentrations of heavy metals, such as Pb, Zn, and Cd [7,19,28,55]. Furthermore, the island is recognized as a “Mediterranean biodiversity hotspot” (15% of the native flora is endemic) [28]. So, investigating these plants is crucial for biodiversity conservation, ecological research, and potential application to phytoremediation. While numerous floristic and vegetational studies have been carried out in abandoned mining sites in Sardinia [19,59,60,61], an update and a comprehensive checklist of Sardinian mining vascular flora is needed. Furthermore, in the last 20 years, a multidisciplinary approach have been used to study species like *Euphorbia pithyusa* L. subsp. *cupanii* (Guss. ex Bertol.) Radcl.-Sm., *Helichrysum microphyllum* Cambess. subsp. *tyrrhenicum* Bacch., Brullo & Giusso, *Juncus acutus* L., *Phragmites australis* (Cav.) Trin. ex Steud., *Pistacia lentiscus* L., *Pinus halepensis* Mill., and *Scrophularia canina* L. subsp. *bicolor* (Sm.) Greuter [5,62,63,64,65]. This approach includes different scientific disciplines such as botany, geochemistry, microbiology, and environmental engineering [7]. Botany can help in floristic and vegetational studies at mining sites by identifying potential tolerant species and applying germination tests under metal(loid)s stress. Geochemistry can provide information about the chemical composition of geochemical spheres and the availability of pollutants and carry out mineralogical investigations of substrates and plant tissues. Microbiology can help in phytoremediation through bioaugmentation and selection of plant growth–promoting bacteria (PGPB). Environmental engineering is fundamental for planning in situ phytoremediation and selecting soil amendments to improve recovery yield. This multidisciplinary approach was also followed in the Iberian Peninsula [9,20,66] and is still under development, with the addition of new tools.

### Aims of This Study

In this study, we present the first inventory of the vascular flora found at abandoned mining sites in Sardinia devoted solely to metal(loid)s exploitation. Compiling a checklist of metallophytes is pivotal in order to set up environmental remediation interventions using phytoremediation activities. Indeed, a deep knowledge of mine flora permits selection of the most suitable plant species, favoring native and endemic *taxa*. The objectives of this study were to: (1) create and present the checklist, summarizing published and unpublished data; (2) provide a list of the metallophytes, classifying them into three categories (obligated, facultative, and occasional) and defining phytostabilizers and phytoextractors for potential remediation activities; and (3) provide information about life forms, chorology, and conservation status, through the development of general and metallophyte-specific biological and chorological spectra.

## 2. Results

The checklist presented here (Table S1, see Supplementary Materials) is composed of 652 *taxa* comprising 510 species and 144 subspecies belonging to 93 families and 355 genera. The most prevalent families were Fabaceae (72 *taxa*; 11%), Asteraceae (64 taxa; 9.8%), and Poaceae (60 *taxa*; 9.2%). Other prevalent families were Orchidaceae, Apiaceae, Brassicaceae, and Lamiaceae with more than 20 *taxa* each. As far as the distribution of genera is concerned, *Trifolium* and *Ophrys* were the most prevalent, with 12 and 11 *taxa*, respectively, followed by *Euphorbia*, *Lotus*, *Galium,* and *Juncus* with 9 *taxa* and *Carex*, *Echium*, *Genista,* and *Lathyrus* with 8 *taxa*.

**Figure 1:**
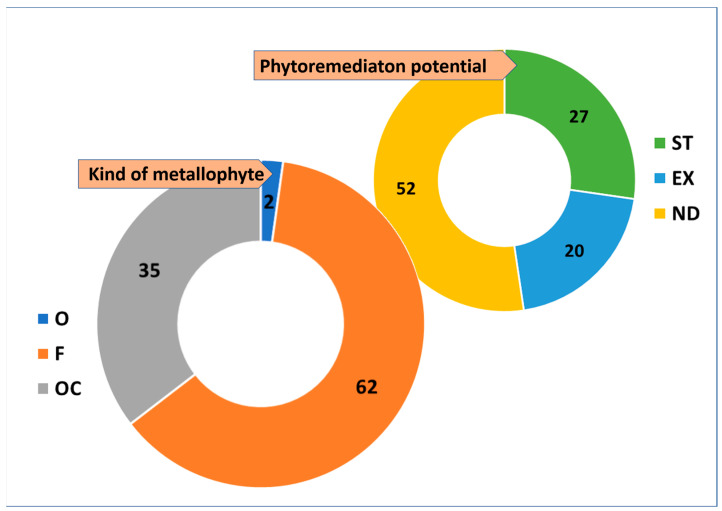
Kinds of metallophytes and their phytoremediation potential. O = obliged (blue); F = facultative (orange); OC = occasional (grey); ST = phytotabilizer (green); EX = phytoextractor (blue); ND = not yet determined (yellow). Data are presented as percentages (%).

As far as metallophyte character is concerned, 319 *taxa* (obligated, O + facultative, F + occasional, OC) out of the total flora (49%) showed this attribute. With regards to the categories of metallophytes identified, facultative metallophytes accounted for 62% (199 *taxa*), while the least common were obligated metallophytes (7 *taxa*; 2%; Figure 1). When phytoremediation potential was considered, 87 *taxa* of metallophytes (27%) are suitable for phytostabilization and 65 for phytoextraction (20%), while 52% of metallophytes have not yet been investigated (Figure 1).

**Figure 2:**
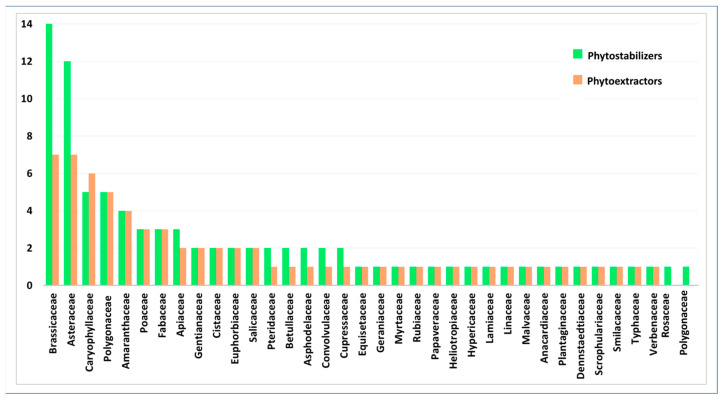
Distribution of phytostabilizers (green) and phytoextractors (orange) among the families.

Fabaceae, Poaceae, Brassicaceae, and Asteraceae were the most prevalent facultative metallophytes, although Brassicaceae and Fabaceae also count as obligated metallophytes, as well as Plumbaginaceae, Linaceae, Primulaceae, and Resedaceae. In detail, among the phytostabilizers, we observed that Poaceae and Fabaceae were predominant, whereas Asteraceae, Brassicaceae, Caryophyllaceae, and Polygonaceae were the most abundant among the phytoextractors (Figure 2).

**Figure 3:**
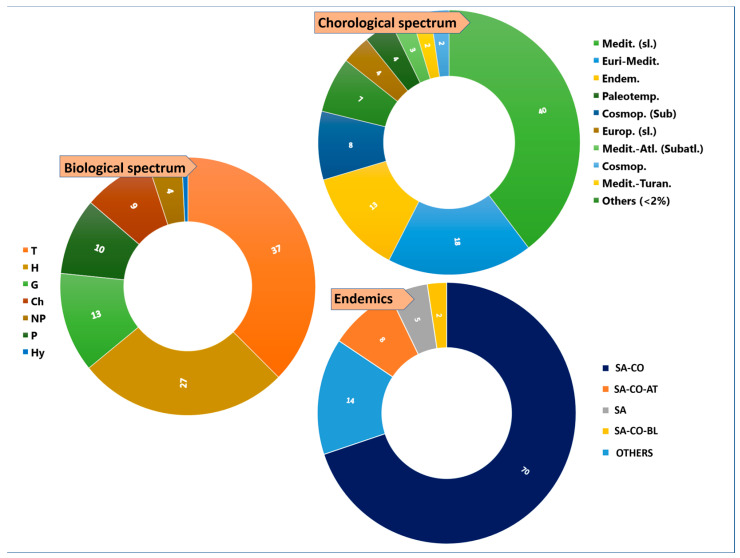
Biological and chorological spectra and details on endemics. T = therophytes; H = hemicryptophytes; G = geophytes; Ch = chamaephytes; NP = nanophanerophytes; P = phanerophytes; Hy = hydrophytes; SA = Sardinia; CO = Corse; BL = Balearic Islands; AT = Tuscan Archipelago. Data are presented as percentages (%).

Analysis of the total flora (Figure 3) showed that the most abundant species were therophytes (T; 37%), followed hemicryptophytes (H; 27%), geophytes (G; 13%), phanerophytes (P; 10%), chamaephytes (Ch; 9%), nanophanerophytes (NP; 4%), and hydrophytes (Hy; 1%).

**Figure 4:**
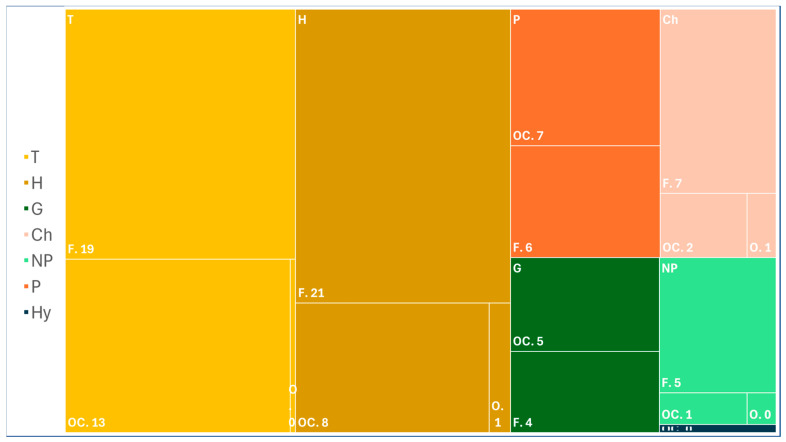
Distribution of metallophytes. F = facultative; O = obliged; OC = occasional; H = hemicryptophytes; Ch = chamaephytes; G = geophytes; NP = nanophanerophytes; P = phanerophytes; T = therophytes. Data are presented as percentages (%).

Figure 4 shows the distribution of the different categories of metallophytes: F metallophytes were mainly present in T, H, Ch, and NP; OC metallophytes were well represented in each life form; and O metallophytes were present only among Ch, NP, and H.

**Figure 5:**
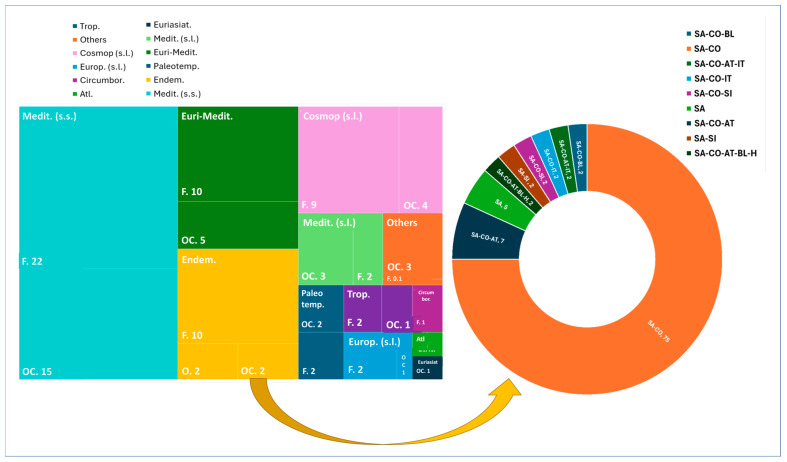
Distribution of metallophytes among chorological forms and details regarding the endemic component. F = facultative; O = obliged; OC = occasional; SA = Sardinia; CO = Corse; BL = Balearic Islands; SI = Sicily; AT = Tuscan Archipelago; IT = Italy. Data are presented as percentages (%).

Chorological data on the total flora (Figure 3) showed a prevalence of Mediterranean *taxa* (40%), followed by Euri-Mediterranean *taxa* (18%) and endemic *taxa* (13%), and these percentages were similar for the metallophytes alone (Figure 5). F and OC metallophytes were well represented in these three chorological forms, while the only obliged observed were endemics (Figure 5). Among the endemics present in the total flora (Figure 3), we observed 82 *taxa* in which Sardinian-Corsican (SA-CO) elements were predominant (70%), followed by Sardinian-Corsican-Tuscan Archipelago elements (SA-CO-AT; 8%) and Sardinian elements (SA; 5%). Minor categories (<2%) were observed in the Others category, which accounted for 7% of the total flora. As far as the metallophytes category is concerned, endemics accounted for 14%, and among them, the SA-CO component was predominant (75%), followed by SA-CO-AT (7%) and SA (5%), and with minor percentages (2% each) of the other components (Figure 5).

**Figure 6:**
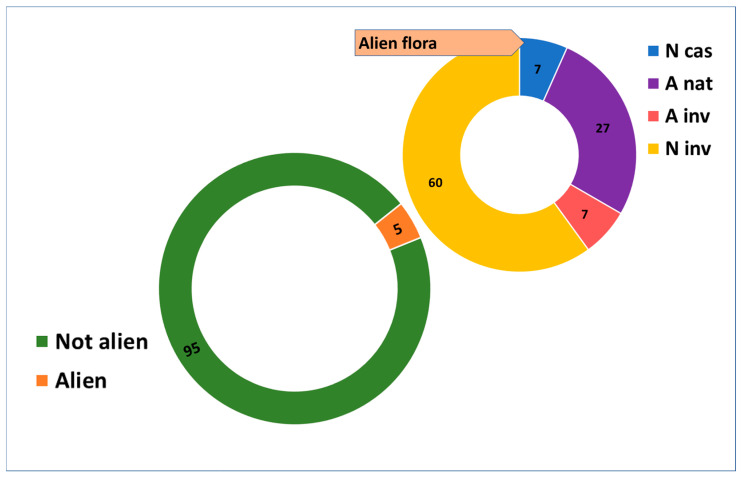
Native and alien flora in mine environments. A = archaeophytes; N = neophytes; cas = casual; nat = naturalized; inv = invasive. Data are presented as percentages (%).

Analysis of the alien component identified 30 alien *taxa* (Figure 6; 5% of the total flora): among them were 20 invasive *taxa* (67%), 8 naturalized (27%), and 2 casual (7%), and the neophytes are predominant towards archaeophytes.

**Figure 7:**
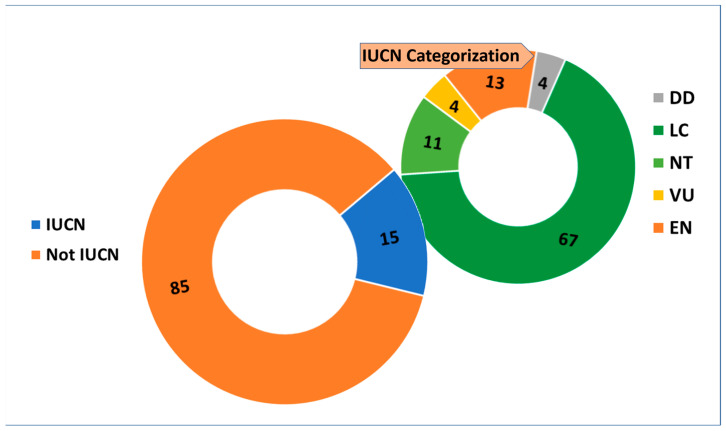
IUCN assessment and details of their risk categories. DD = Data deficient; LC = Least concern; NT = Near threatened; VU = Vulnerable; EN = Endangered. Data are presented as percentages (%).

From a conservation point of view, only 15% (98 *taxa*) of the total flora is included on the Italian Red List: 67% of these *taxa* are classified as Least concern (LC), followed by Endangered (EN) at 13%, Near threatened (NT) at 11%, Vulnerable (VU; 4%), and Data deficient (DD; 4%, Figure 7).

**Figure 8:**
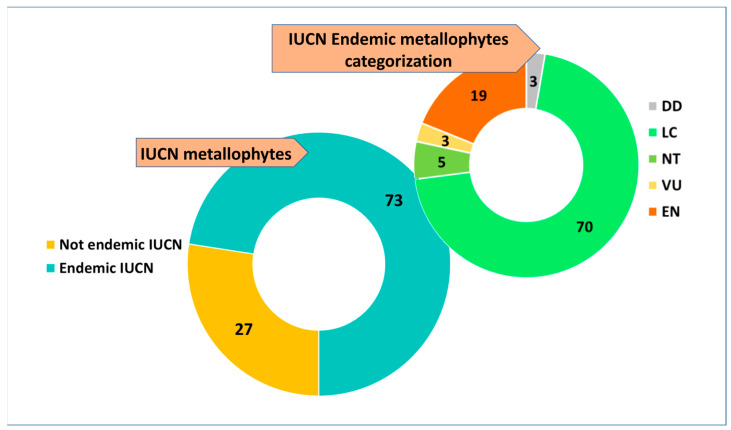
IUCN assessment of metallophytes and details of endemic risk categories. DD = Data deficient; LC = Least concern; NT = Near threatened; VU = Vulnerable; EN = Endangered. Data are presented as percentages (%).

If only the metallophyte category is considered, the 16% (51 *taxa*) are included on the Italian Red List. Among the IUCN-listed metallophytes, 73% (37 *taxa*) are endemics, of which 70% are LC, followed by 19% EN, 5% NT, and 3% VU and DD (Figure 8).

**Figure 9:**
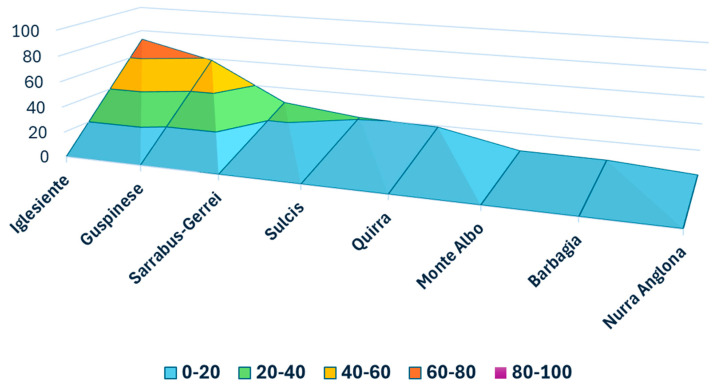
Distribution of metallophytes among different mine areas in Sardinia. Data are presented as percentages (%).

As far as the distribution of metallophytes among the different mine areas of Sardinia is concerned, the most represented was Iglesiente (72% of metallophytes), followed by Guspinese (59%) and Sarrabus (28%; Figure 9).

## 3. Discussion

Analysis of the distribution among families showed that the most abundant were Fabaceae, Poaceae, and Asteraceae, in agreement with data reported for the Iglesiente Guspinese, Sarrabus-Gerrei, and Quirra mining districts [5,19,59]. Orchidaceae immediately followed the abovementioned families, and their relative abundance was not a surprise. Indeed, there is evidence that, in the Mediterranean bioclimate, calcareous and serpentine substrates are common favorable conditions for orchids in Sardinia in the Iglesiente mine district [67] and Barbagia [68], as well as elsewhere like in the Balkans [69]. As far as the metallophyte character of the investigated flora is concerned, the results showed a high presence (49%) of *taxa* with this characteristic. Inside this cluster, the high percentage of F (62%) showed that many plant species have a survival/adaptation mechanism to the stressful conditions of mine waste materials like the absence of a topsoil, lack of nutrients and organic matter, and high concentration of metal(loid)s [7].

On the other hand, we found also a not negligible percentage of O metallophytes (2%), which shows the presence of extremely adapted endemic species to these unfavorable conditions with a strictness distribution of few km^2^, like *Linum mulleri* Moris, *Limonium merxmuelleri* Erben subsp. *merxmuelleri*, *Genista insularis* Bacch., Brullo & Feoli Chiapella subsp. *fodinae* Bacch., Brullo & Feoli Chiapella, and *Centranthus pontecorvi* Bacchetta & Brullo. However, 36% of the assessed metallophytes were OC, showing again that, even if they are not common in these environments, they are resilient to the stressful conditions of mining wastes. In our opinion, the Sardinian mining flora reflects the broad concept of edaphism. Indeed, the observed flora and vegetation followed many of the points noted by Mota et al. [32], such as: (i) the presence of characteristic species, some of them endemic and living only in these types of substrates; and (ii) sharp discontinuities with the surrounding vegetation, identifiable by physiognomic features. Moreover, also the presence of many edapho-physical-chemical factors that determine edaphism [32] were detected, like the lack of nutrients and organic matter, high concentrations of metals, the texture of mine substrate, the instability of the substrates on slopes, the presence of slow and poor biological processes and pedogenesis, and plant–plant interactions (i.e., nursery species). While other edaphism cases are directly linked to specific priority habitats by the EU Habitats Directive, like the *Gypsophiletalia* order [70], this is not the case for the Sardinian region. Indeed, Fois et al. [61] proposed an improvement to Annex I of Directive 92/43/EEC with the new habitat “Calaminarian vegetation of mining dumps, tailing dams and quarries”. In comparison with other plant communities typical of mining environments, in Europe these species are grouped in the *Violetalia calaminariae* order [71]. In Western-central and Western Europe, *Thlaspion calaminariae* alliance is common in heavy-metal soils [72], while in Central Europe *Armerion halleri* alliance is prevalent [71].

Within this framework, the predominance of Fabaceae, Asteraceae, and Poaceae as phytostabilizers and Asteraceae and Brassicaceae as phytoextractors (Figure 2) is common and has already been observed in different mine districts of Sardinia [59,60,73] and at other sites around the Mediterranean Basin [7]. These families are recognized as being in *taxa* with high levels of metal tolerance [7,74,75,76], and in many cases of accumulators and hyperaccumulators and being also the most representative families in the Mediterranean floras [77]. It is also important to highlight that the most common families among phytoextractors are often species that are highly palatable to humans (e.g., thistles, chard, and spinach) and farm animals, and this aspect can represent an important health problem.

Without a doubt, metallophytes must be considered primary when phytoremediation activities are planned, and their potential must be known. From our results, it appears that 27% of metallophytes are suitable for phytostabilization (ST) and for long-term rehabilitation of these sites. Indeed, these *taxa*, which exclude metal(loid)s in their roots or in the rhizosphere, limit the dispersion of contaminants, favoring the recovery of the natural vegetation dynamics and the establishment of a durable plant canopy. Regardless, 20% of the metallophytes were found to be suitable for phytoextraction (EX) and for the recovery of metals. These *taxa* have a greater potential of accumulation in epigeal organs and can be useful for the recovery of metals, but phytoextraction and phytomining must be carefully considered in terms of their intrinsic weaknesses, like the negative influence on biodiversity due to the extensive use of monotypic plantings, the disposal of harvested hazardous plants, and the risk of phytoextracts entering the food chain [7]. Furthermore, 52% of the metallophytes we identified have still not been investigated (ND), and assessing their phytoremediation potential is of noteworthy importance.

Analysis of the distribution among life forms highlighted a high presence of T and H in these kinds of environments that is linked to habitat degradation, although this value was lower than that in more disturbed environments like urban and overgrazed-trampled environments [51,52]. Moreover, the T and H abundance was in agreement with those observed in a single mine district in Sardinia [59,60,73]. T species are synanthropic species, common to degraded and altered habitats. In our case, T are predominant in mine dumps and mine wastes not already consolidated from a granulometric point of view and with a high concentration of heavy metals [19]. Therophytes create annual meadows that can evolve as the concentration of metals decreases and pedogenesis starts. Moreover, the absence of obligated metallophytes among therophytes indicates that, even if ephemeral species have not specialized to colonize contaminated substrates, they can tolerate the presence of toxic elements which may accumulate in their short life cycle, allowing them to bloom and disperse seeds. Despite the habitat and soil degradation with high concentrations of metal(loid)s in these environments, P and NP species showed percentages similar to those observed by Bacchetta et al. [55] at the Montevecchio mine sites (SW Sardinia) and by Pontecorvo at the Iglesiente mine sites and in more natural contexts [6,78], but higher than those observed by Iiriti [59] for the Sarrabus-Gerrei and Quirra districts. This can be explained by the frequent proximity of mining areas to woodland formations and scrublands of medium-high naturalness, which can spread inside mining sites in a relatively short time. Indeed, several plant coenoses comprising NP and P have been described, like *Euphorbio cupanii-Santolinetum insularis* Angiolini & Bacchetta 2003 or *Dorycnio suffruticosi-Genistetum corsicae* Angiolini, Bacchetta, Brullo, Casti, Giusso & Guarino, 2005 [19], which colonized old and well-consolidated mining dumps. Moreover, the high percentage of H can be correlated with the abundance of natural rocky crevices and Mediterranean climatic conditions [68]. Indeed, in incoherent mine dumps with high granulometry, similar ecological conditions of rocky crevices and torrential regime riverbed can occur. G species are typically common in areas outside and surrounding mine areas and have good adaptability to poor substrates; this life form is the third most abundant, confirming its high adaptability to some human disturbances, like overtrampling, vegetation degradation, woodland pastural activity, and fires, which are very common in Sardinia and in mine areas [70,79]. The G value reported here is similar to those observed in other mine districts in Sardinia [59,60,73]. As far as Hy species are concerned, although their percentage was low (1%), their ecological role in these environments is pivotal. The small percentage of hydrophytes, including some generally common ones such as *Lemna* spp., confirmed their high susceptibility to water contamination, which enables them to serve as useful bio-indicators [80,81]. However, mining environments contain quarry and mining ponds created by excavation activities. In many cases, they are considered a disservice to the ecosystem and a threat to human health and wildlife due to the polluted water. Once abandoned, they can be revegetated naturally by some pollution-resistant hydrophytes and other wetland plants, such as *Typha* spp. or *Phragmites australis* (Cav.) Trin. ex Steud., which provides an ecosystem service by purifying water and providing new habitats. If the distribution of metallophytes among life forms is taken into consideration (Figure 4), OC species are widespread among all life forms, suggesting that species with varying functional traits may be adapted to different ecological conditions; F species mainly presented as T, H, and Ch, confirming the typicality of such life forms at mine sites; and O species were distributed in a few categories (Ch, NP and H), showing their high rate of extreme adaptation to restrictive environments [60].

Considering chorological distribution, the Mediterranean and Euri-Mediterranean components were dominant, as has also been observed in other Sardinian mining floras [59,60,73]. Moreover, the Mediterranean character of the area was confirmed by the H/T index = 0.7 (T = 37%; H = 27%), as proposed by Cannucci et al. [82], where typical Mediterranean conditions occur if the H/T ratio is < 1. An important presence in terms of endemic *taxa* (13%) was found: this value can be explained by the generally high rate of Sardinian endemics (15% of the native flora) [28] and by the presence of very peculiar growing conditions. Some of the endemic *taxa* can be considered a case study in metal edaphism; in particular, *L. mulleri* and *L. merxmuelleri* subsp. *merxmuelleri*, whose habitats are strictly linked to metal-enriched substrates, behaving as obliged metallophytes. Also, other Mediterranean mining sites exhibit a large number of endemic species with metallophytic or serpentinophytic character, for instance *E. andevalensis* (Iberian Pyrite Belt, Spain and Portugal), different species of *Onosma* and *Alyssum* [23,24], and *Odontarrhena stridii* L. Cecchi, Španiel & Selvi [24] in Greece. The largest portion of the endemic flora is composed by *taxa* shared with Corsica (SA-CO), together with those shared with the Tuscan Arcipelago (SA-CO-AT), which are consistent with other Sardinian mining floras [59,60,73] and reflect the geological events that occurred in these areas. Indeed, Sardinia and Corsica are part of the Cyrno-Sardinian microplate that split apart from the current Gulf of Lion (S France) in the Oligocene and were intermittently connected until the Pleistocene glaciations [83,84,85,86]. During the same Plio-Pleistocene eustatic fluctuations, a land bridge connected the Italian Peninsula to Corsica and Sardinia through the Tuscan Archipelago [28]. Other minor components, like endemic *taxa* shared with Balearic Islands (2.4%), are also mainly explainable by the geological history until the Oligocene, as being part of the same Proto-Hercynian Ligurian massif [84]. Despite being the richest Sardinian endemic form, SA species were less frequent in mines: this is because SAs are generally concentrated in coastal and high mountain environments, where mining activities are uncommon [28]. Moreover, SA species are generally linked to carbonatic substrates, which are less present in mine areas (with few exceptions), whereas SA-CO and SA-CO-AT are more common in silicate substrates, which are typical of mining areas.

Although they represent a minority, alien species accounted for approximately 5% of the flora, primarily invasive species (neophytes and archaeophytes). While this low percentage reflects a high ecological value, it also raises concerns, posing a threat to local biodiversity. Notably, two identified species—*Acacia saligna* (Labill.) H.L. Wendl. and *Ailanthus altissima* (Mill.) Swingles—were classified as alien species of European concern under Regulation EU 1143/2014. Despite their metallophyte characteristics, which make them suitable for phytoremediation, their use should be avoided due to their potential negative impacts on local ecosystems and human well-being. Accordingly, it is widely recommended to prioritize the use of native species over non-native or alien species for phytoremediation efforts [46]. Nowadays, no efforts in terms of invasive alien species (IAS) eradication or mitigation in these environments are carried out; however, some methodologies have been proposed and applied in other contexts, for example the eradication of *A. saligna* from dunes and coastal habitats [87]. These methods can be tailored to mine environments and thus applied in these contexts.

From a conservation perspective, 15% of the total flora has been assessed based on IUCN criteria. Of this group, a moderate proportion has a threat category (EN, VU, NT; 29%), with 13% considered endangered (EN). If endemic *taxa* categorized as metallophytes are taken into account, 73% (37 *taxa*) are categorized on the Italian Red List, with 19% considered EN, like *Dianthus cyathophorus* Moris subsp. *cyathophorus*, *Genista sardoa* Vals., *Hypericum scruglii* Bacch., Brullo & Salmeri, and *L*. *mulleri*, and hold particular conservation significance due to their endemic status, conservation importance, and metallophyte characteristics. Nevertheless, *L. mulleri* is also included as a priority *taxon* of the Habitat Directive (92/43/ECC). Often, the main threat to these *taxa* is the fragility of the populations and of their habitat. Moreover, the narrow ecological range and their insulation represent risk factors for their persistence. A further threat is represented by environmental restoration of disused mining landfills using disruptive methods like excavation, which could lead to a decline in the availability and quality of the habitat suitable for the *taxa*.

The use of native and endemic species is compatible with the ecological, climatic, and soil conditions because they are already adapted to these environments [7]. Furthermore, it is suggested to avoid the use of IAS, as they would threaten local biodiversity.

If the distribution of metallophytes among the different Sardinia’s mine sites is take into consideration, Guspinese-Iglesiente and Sarrabus Gerrei mine sites are more represented than at others; however, it is important to highlight that these districts are more studied than others (i.e., Monte Albo, Barbagia).

From an economic point of view, phytoremediation is more advantageous than conventional techniques (mechanical excavation, etc.): it is sustainable, eco-friendly, and an efficient alternative to conventional methods [88].

## 4. Materials and Methods

### 4.1. Study Area

Sardinia is the second largest Mediterranean island (total surface area of 24,090 km^2^). The Sardinian landscape is heterogenous, with hills, plateaus, plains, and several isolated groups of low mountains or massifs [28]. This heterogeneity is reflected in the substrata: Palaeozoic limestone, metamorphites and batholiths, passing through a sedimentary lithostratigraphic complex of the Mesozoic, Tertiary marine and volcanic depositions, and Quaternary alluvial deposits [28]. There are two macrobioclimates (Mediterranean and temperate subMediterranean), eight thermotypic horizons (from lower thermoMediterranean to upper supratemperate), and seven ombrothermic horizons (from lower dry to lower hyperhumid) [28,89].

**Figure 10:**
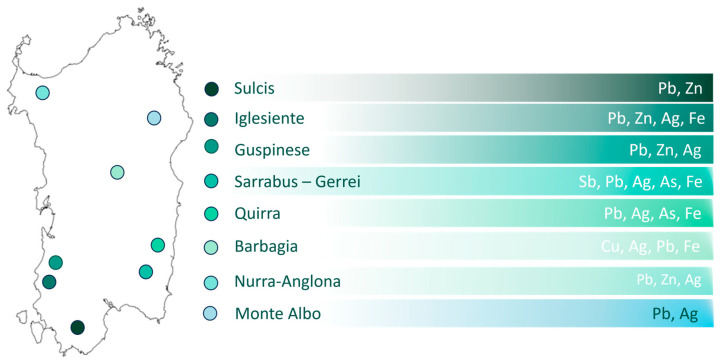
Maps of the main mine areas in Sardinia and the most commonly extracted metal(loid)s.

Sardinia was historically devoted to mining activities since the Bronze and Early Iron Ages during the Nuragic period [26]. Intensive exploitation started during the industrial period, in the second part of XIX century [7,90]. Mining activities mainly ceased in 1990s, especially because of competition with mines in other countries. However, hundreds of mining waste dumps exist, with millions of tons of polluted materials left to weathering and dispersal, affecting terrestrial and aquatic ecosystems, as well as human health [2,62,91]. Abandoned mine sites are spread all over the island, but the most important sites in terms of time of activity and extension are Sulcis-Iglesiente and Guspinese-Arburese (SW Sardinia), Sarrabus-Gerrei and Quirra (SE Sardinia), Barbagia (Centre Sardinia), Nurra-Anglona (NW Sardinia), and Monte Albo (NE Sardinia), as shown in Figure 10. The most extracted metals were Zn, Pb, Cd, Ag, Fe, and Sb, but some differences in terms of geochemistry characteristics are recognized in each sector. The substrate coming from Sulcis generally derived from orthogneiss during the Ordovician period, while those from Iglesiente derive from carbonate formations from the Palaeozoic period (mainly from the so called “Metalliferous”) and are rich in terms of Zn, Cd, and Pb mineralization [92]. As far as the substrates of Guspinese mine sites are concerned, these derived from a small lens of metamorphic rocks rich in Zn, Pb, and Ag originating from the Arburese batholith [93,94]. Mine wastes in this area undergo oxidation reactions accompanied by the release of metals. As a result, an extremely acidic environment is produced (pH between 2 and 4), with further dissolution of other sulfides [95]. The mineralization at Sarrabus-Gerrei and Quirra derives from stratabound rocks of the Ordovician and Devonian periods and from sulfides (barite, fluorite) [59]. In the Nurra-Anglona sector, mineralization is linked to vulcano-sedimentary oolit iron lenses and hydrothermal Pb-Zn– and Sb-bearing veins [96]. Considering the ore deposit of Barbagia, and in particular for “Funtana Raminosa”, they are generally hosted in hydrothermal rocks, with some skarn [97]. The Monte Albo sector is characterized by Jurassic limestone formations (limestones and dolomites of Monte Albo) and by Hercynian schists and plutonites. In addition to these lithologies, there are various vein manifestations, predominantly quartzose, mineralized with Pb, Ag, Zn, and fluorite [92]. However, the extraction of these metals was accompanied also by other harmful metal(loid)s (e.g., As), causing a health hazard and a serious case of environmental pollution [2,98,99,100]. Moreover, since cessation of exploitation, very few remediation actions have been designed and implemented [7]; hence, huge quantities of polluted materials were left abandoned (c.a. 70 Mm^3^). In Decree No. 334/1999, the Italian Government declared the mineralized areas of Sardinia as high-risk zones for environmental crises and potential threats to public health. From 2000 to 2024, many floristic and vegetational studies were carried out, highlighting the presence of numerous endemic *taxa* with phytogeographic interest, like *Echium anchusoides* Bacch., Brullo & Selvi, *Galium schmidii* Arrigoni, *Helichrysum microphyllum* Cambess. subsp. *tyrrhenicum* Bacch., Brullo & Giusso, *Iberis integerrima* Moris, *Linum mulleri* Moris, *Ptilostemon casabonae* (L.) Greuter, *Santolina corsica* Jord. & Fourr., *Polygala padulae* Arrigoni, *Reseda luteola* L. subsp. *dimerocarpa* (Müll.Arg.) Abdallah & de Wit, and *Lysimachia monelli* (L.) U. Manns & Anderb. In addition, the presence of plant assemblages peculiar to these environments, such as *Coincyo recurvatae-Helichrysetum microphylli Angiolini, Bacchetta, Brullo, Casti, Giusso & Guarino, Resedo luteolae-Limonietum merxmuelleri* Angiolini, Bacchetta, Brullo, Casti, Giusso Del Galdo & Guarino, or “the Sardinian special series of heavy metal–polluted mine substrates” were recognized [19,60]. With the end of mining activity, landfills and tailings basins were colonized by herbaceous communities. Substrates derived from mining activities, even before the onset of pedogenetic processes, were colonized mainly by ephemeral meadows, composed of therophytes on silty-clayey substrates (Aggr. of *Centaurium erythraea* Rafn and *Bellium bellidioides* L.) and on gravelly slopes with little coherence with high concentrations of heavy metals (Aggr. of *Jasione montana* L. and *Rumex bucephalophorus* L.). Whilst, on incoherent substrates consisting of coarse material, chamaephytic and hemicryptophytic vegetation are typical [43]. These garrigues are particularly interesting from a biogeographical point of view because they are rich in endemic species like *P. casabonae*, *E. cupanii*, *L. merxmulleri* subsp. *merxmuelleri*, *I. integerrima*, *E. anchusoides*, and *S. canina* subsp. *bicolor.* For this reason, Angiolini [19] proposed a new Sardinian-Corsican endemic alliance (*Ptilostemono casabonae-Euphorbion cupanii* Angiolini, Bacchetta, Brullo, Casti, Giusso Del Galdo & Guarino). Moving forward with vegetational evolution, low maquis, present only in landfills abandoned for several years that have been well consolidated, is characterized by a predominance of *Genista corsica* (Loisel.) DC. (*Dorycnio suffruticosi-Genistetum corsica* Angiolini, Bacchetta, Brullo, Casti, Giusso & Guarino). Finally, progressive evolution of the soil leads over time to the establishment of species and communities typical of uncontaminated environments [43].

### 4.2. Data Collection

The floristic checklist presented here was derived from a broad analysis of the literature concerning floristic and vegetational analyses of Sardinian mining areas (only areas devoted to metal(loid)s extraction), unpublished data from different field surveys carried out from 2000 to 2024 within the framework of a different project that our research group was involved in (i.e., germplasm collection, habitat monitoring), and analysis of CAG, SS, and SASSA herbaria exsiccata. It is undeniable that, over the years, some areas (for example Sulcis-Iglesiente) have been studied more than others, and therefore this aspect can lead to overestimation. However, since this is a checklist only, the presence of a certain *taxa* has been considered, and not the abundance in each place. Plant nomenclature follows Bartolucci et al. [101] and Galasso et al. [102] for native and alien plants, respectively. Family names follow PPG I [103] for pteridophytes, Pignatti et al. [104] for gymnosperms, and APG IV [105] for angiosperms. Life forms were assigned following Raunkiaer’s classification [106], whereas chorology follows the abbreviations proposed by Pignatti et al. [104]. Alien categorizations were made based on the national standardized system [102] basing on the definition of Pyšek et al. [107]. An alien *taxon* is defined as a plant whose presence can be ascribed to intentional or unintentional anthropogenic activities or to natural spread from the native area. A casual *taxon* is an alien plant that can bloom and occasionally produce offspring beyond cultivation or for unintended reasons. Regardless, persistence is limited because it is unable to establish self-sustaining populations. Naturalized alien plants generate self-maintaining populations without direct human intervention, whereas invasive plants produce fertile offspring at considerable distances and are able to spread in a large area without control. Alien plants generate self-maintaining populations without direct human intervention, produce fertile offspring at considerable distances from the parent individuals, and are able to spread over a large area. We also distinguished archaeophyte *taxa,* which are alien plant introduced to Europe before 1492, from neophyte *taxa* introduced after 1492. In order to assess conservation status, IUCN categories were assigned following the most recent Italian Red List [108].

The detected *taxa* were categorized into three metallophyte categories: obliged (O), facultative (F), and occasional (OC). This categorization was performed based on distribution along the island and presence on metal(loid)-polluted or naturally enriched substrates. Obliged metallophytes (O) are here defined as *taxa* present only on substrates polluted in metal(loid)s or on natural metal-enriched sites. Facultative metallophytes (F) are *taxa* able to grow in both metal-polluted/enriched and unpolluted substrates. Considering that some *taxa* seem to be uncommon at mining sites due to the extremely high concentration of metals, but are at times present anyway (i.e.,: *Pistacia lentiscus* L., *Quercus ilex* L.) and often have shown phytoremediation potential [7], in this study we created a third category of metallophytes in order to categorize this behavior. These *taxa* were defined as occasional (OC), indicating that they are present at mine sites but are generally uncommon in polluted/metal-enriched substrates. *Taxa* that did not fall into one of these three categories, even though they are present in mine environments, were rare (casual), so we do not include them in the calculations concerning metallophytes. Our proposed classification of a metallophyte based on the presence/absence of a *taxon* on polluted/unpolluted substrates is compatible to that proposed by Baker [109], which considered the metal survival strategy. For a better understanding of the terminology used, definitions related to metallophytes and phytoremediation are included in Table A1 (Appendix A).

Categorization of *taxa* as phytostabilizers or phytoextractors was performed based on the published literature as of 2024 (see Table S1), using biological indices used to estimate accumulation in plant tissue. The most common indices used for the estimation of phytoremediation potential are the Biological Concentration Factor (BCF) [110], the Biological Accumulation Coefficient (BAC) [111], and the Translocation Factor (TF) [110].

## 5. Conclusions

The checklist of the mining vascular flora in Sardinia shows the presence of abundant biodiversity, despite the restrictive environmental conditions common tomining environments. A large number of endemics with a very limited distribution were recognized, showing a high level of specialization of certain *taxa* (e.g., *L. merxmuelleri* subsp. *merxmuelleri*, *L. mulleri*, and *G. insularis* subsp. *fodinae*), as well as the presence of numerous endangered *taxa*. The presence of numerous obliged metallophytes demonstrated the presence of very peculiar flora that must be deeply investigated for future phytoremediation. Hence, a deep knowledge of the local flora of mine environments, including metallophytes and their suitability for phytoremediation, can help in the design of more sustainable phytoremediation approaches. Within this framework, when an unstudied *taxon* is chosen for deeper phytoremediation study, a multidisciplinary approach is desirable, as already shown in numerous studies. Furthermore, some metallophytes and their habitats must be better protected, as demonstrated by the presence of numerous endangered vascular plants. Last but not least, our work represents an initial inventory that can be expanded over time, adding new species and better investigating less explored mine sites in Sardinia.

## Data Availability

Data are contained within the article and Supplementary Materials.

## Supplementary Materials

The following supporting information can be downloaded at: https://www.mdpi.com/article/10.3390/plants14081225/s1, Table S1: list of *taxa* of Sardinia metallophytes. Refs. [112,113,114,115,116,117,118,119,120,121,122,123,124,125,126,127,128,129,130,131,132,133,134,135,136,137,138,139,140,141,142,143,144,145,146,147,148,149,150,151,152,153,154,155,156,157,158,159,160,161,162,163,164,165,166,167,168,169,170,171,172,173,174,175,176,177,178,179,180,181,182,183,184,185,186,187,188,189,190,191,192,193,194,195,196,197,198,199,200,201,202,203,204,205,206,207,208,209] are cited in the Supplementary Materials.

## Appendix A

**Table A1:** Glossary of terminology related to metallophytes and phytoremediation.

	Definition
Accumulator species	*Taxa* where metals are highly translocated and accumulate in epigeal organs. Suitable for phytoextraction.
Excluder species	*Taxa* that exclude metal(loid)s in the rhizosphere region and prevent their translocation into epigeal organs. Suitable for phytostabilization.
Facultative metallophyte	*Taxa* able to grow in both metal-polluted/enriched and unpolluted substrates.
Metallophytes	Plants growing on metal(loid)-polluted substrates or substrates naturally enriched in metal(loid)s that have developed intrinsic resilience to metal(loid) stress.
Obligate metallophytes	*Taxa* that can live and thrive only on metal(loid)-polluted substrates or substrates naturally enriched in metal(loid)s.
Occasional metallophytes	*Taxa* that are present at mining sites but are generally uncommon in polluted/metal-enriched substrates.
Phytoextractors	*Taxa* that are suitable for phytoextraction. They are generally metal accumulator and hyperaccumulator species.
Phytoextraction	Application of phytoremediation devoted to the economic recovery of metals from substrates.
Phytoremediation	Technology by which vascular plant species and their associated microbiota, in combination with amendments and different kind of agronomic strategies, are used to remove or limit contamination or make it as harmless as possible.
Phytostabilization	Application of phytoremediation suitable for stabilizing mine substrates from weathering, for creating a long-term plant canopy, and for reducing the visual impact of excavation and mine waste accumulation in dumps.
Phytostabilizer	*Taxa* that are suitable for phytostabilization. They are generally metal excluder species.
